# Outcome of *ABCA4* microarray screening in routine clinical practice

**Published:** 2009-12-20

**Authors:** Paul J.G. Ernest, Camiel J.F. Boon, B. Jeroen Klevering, Lies H. Hoefsloot, Carel B. Hoyng

**Affiliations:** 1 Department of Ophthalmology, University Hospital Maastricht, Maastricht, The Netherlands; 2 Department of Epidemiology, Maastricht University, Maastricht, The Netherlands; 3 Department of Ophthalmology, Radboud University Nijmegen Medical Centre, Nijmegen, The Netherlands; 4 Department of Human Genetics, Radboud University Nijmegen Medical Centre, Nijmegen, The Netherlands

## Abstract

**Purpose:**

To retrospectively analyze the clinical characteristics of patients who were screened for mutations with the ATP-binding cassette transporter gene *ABCA4 (ABCA4)* microarray in a routine clinical DNA diagnostics setting.

**Methods:**

We performed a retrospective analysis of the medical charts of 65 patients who underwent an *ABCA4* microarray screening between the years 2002 and 2006. An additional denaturing gradient gel electrophoresis (DGGE) was performed in these patients if less than two mutations were found with the microarray. We included all patients who were suspected of autosomal recessive Stargardt disease (STGD1), autosomal recessive cone–rod dystrophy (arCRD), or autosomal recessive retinitis pigmentosa at the time of microarray request. After a retrospective analysis of the clinical characteristics, the patients who were suspected of STGD1 were categorized as having either a typical or atypical form of STGD1, according to the age at onset, fundus appearance, fluorescein angiography, and electroretinography. The occurrence of typical clinical features for STGD1 was compared between patients with different numbers of discovered mutations.

**Results:**

Of the 44 patients who were suspected of STGD1, 26 patients (59%) had sufficient data available for a classification in either typical (six patients; 23%) or atypical (20 patients; 77%) STGD1. In the suspected STGD1 group, 59% of all expected pathogenic alleles were found with the *ABCA4* microarray. DGGE led to the finding of 12 more mutations, resulting in an overall detection rate of 73%. Thirty-one percent of patients with two or three discovered *ABCA4* mutations met all typical STGD1 criteria. An age at onset younger than 25 years and a dark choroid on fluorescein angiography were the most predictive clinical features to find *ABCA4* mutations in patients suspected of STGD1. In 18 patients suspected of arCRD, microarray screening detected 22% of the possible pathogenic alleles.

**Conclusions:**

In addition to confirmation of the diagnosis in typical STGD1, *ABCA4* microarray screening is usually requested in daily clinical practice to strengthen the diagnosis when the disease is atypical. This study supports the view that the efficiency and accuracy of *ABCA4* microarray screening are directly dependent upon the clinical features of the patients who are screened.

## Introduction

The autosomal recessive form of Stargardt disease (STGD1) is caused by variations in the ATP-binding cassette transporter gene *ABCA4 (ABCA4)* [1]. The gene product of *ABCA4* is localized to the rim of the rod and cone outer segment disc membranes [2,3]. Pathological mutations in *ABCA4* result in an accumulation of all-trans retinal in the membrane of the photoreceptor disc. Following phagocytosis of the photoreceptor outer segments, toxic derivatives of all-trans retinal accumulate in the retinal pigment epithelium (RPE) and may ultimately lead to RPE cell death and atrophy of the overlying photoreceptor layer [4]. Besides STGD1, *ABCA4* mutations may also be found in a significant percentage (65%) of patients with autosomal recessive cone–rod dystrophy (arCRD) [5]. In addition, mutations in this gene have been described in atypical autosomal recessive retinitis pigmentosa (arRP) and may play a role in age-related macular degeneration [6,7].

STGD1 appears to be monogenic; no sibships have been found in which *ABCA4* alleles do not co-segregate with the disease. However, intrafamilial variability of the phenotype has been reported [8]. Differences between siblings may indicate the influence of environmental factors as well as other genes that modify the expression of a given *ABCA4* genotype. Moreover, *ABCA4* has a large allelic diversity. The nucleotide diversity of the *ABCA4* coding region was found to be 9–400 times greater than that of two other macular disease genes that were examined in a similar fashion (*bestrophin 1* and *EGF-containing fibulin-like extracellular matrix protein 1*) [9].

Allelic heterogeneity and the size of the gene have substantially complicated genetic analysis of *ABCA4*-associated retinal disease. All mutation detection techniques that remain exclusively PCR-based are relatively inefficient, expensive, and labor intensive. Therefore, the *ABCA4* genotyping microarray was developed [10]. The ABCR400 microarray contains all disease-associated genetic variants and many polymorphisms of the *ABCA4* gene that have been reported in the peer-reviewed literature or that have been directly reported to Asper Biotech, the producer of the microarray. In 2003, Jaakson and coworkers [10] found a mutation allelic detection rate of 55%–65% in patients with STGD1 by using the microarray alone. In combination with a conventional mutation detection method, for example, single-strand conformation polymorphism (SSCP) technology, a detection rate of about 70%–75% may be achieved.

This study evaluated the *ABCA4* microarray in a routine clinical DNA diagnostics setting by performing a retrospective analysis of the clinical characteristics of patients who were screened with the microarray.

## Methods

Sixty-five patients who underwent an *ABCA4* microarray screening between the years 2002 and 2006 at the Department of Human Genetics, Radboud University Nijmegen Medical Centre (Nijmegen, The Netherlands), were included in this retrospective study. An *ABCA4* microarray screening was always requested by an ophthalmologist who saw the patient at the outpatient department of our hospital. The most likely clinical diagnosis, judged by the ophthalmologist, was noted at the time of request. Information on family history, taking into account hereditary eye diseases, was routinely obtained before the genetic screening was requested. We included all patients who were suspected of STGD1, arCRD, or arRP, so no dominant inheritance pattern was seen in the families of these patients. We had no influence on the decision making of the clinicians who requested the microarray screening. Only probands were included. Patients from families in which we had already found *ABCA4* mutations were excluded along with patients whose clinical information was not available. This study was approved by the local ethics committee and all participants gave informed consent for using their data, in accordance with the tenets of the Declaration of Helsinki.

Peripheral venous blood samples were obtained and the genomic DNA was isolated from leukocytes for analysis according to standard methods using salt extraction. Microarray screening was performed by Asper Biotech (Tartu, Estonia) according to a previously described method [10]. If less than two mutations were found, an additional denaturing gradient gel electrophoresis (DGGE) was performed according to a previously described method, using a gradient of 10% to 65% [11]. Primers were chosen to surround all exons (primer sequences are available on request). All mutations that were found by the chip or by DGGE were confirmed by direct sequencing. The pathogenicity of a certain mutation was determined by our own research protocol based on the criteria described by Cotton et al. [12]. Taken into account were for instance the evolutionary conservation of an amino acid, the kind of change (Grantham score), and information from the online prediction programs SIFT and Polyphen. The occurrence of novel variants was analyzed by sequence analysis in 100 control individuals from an earlier study [13]. Mutation numbering throughout the text and tables is based on the cDNA sequence (GenBank NM_000350). If available, genetic information of family members was used for segregation analysis. The effectiveness of the chip was determined for each clinical indication by determining which percentage of total expected pathogenic alleles was found.

**Figure 1:**
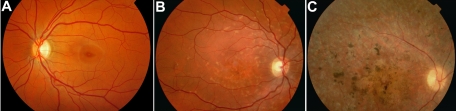
Fundus appearances according to Fishman’s three-class system [14]. **A**: This photograph represents fundus appearance type I, which is characterized by a small atrophic-appearing foveal lesion. This lesion can be surrounded by parafoveal or perifoveal yellowish white lesions. **B**: This photograph represents fundus appearance type II, showing numerous yellowish white fundus lesions extending beyond the vascular arcades. **C**: This photograph represents fundus appearance type III, which is characterized by extensive atrophic-appearing changes of the retinal pigment epithelium throughout the posterior pole, extending beyond the vascular arcades.

We collected the age at onset, the best corrected visual acuity (BCVA), fundus appearance according to Fishman’s three-class system [14] (group I is characterized by disease confined to the macula, group II by any flecks peripheral to the temporal vascular arcades, whereas group III is characterized by chorioretinal and/or RPE atrophy, and/or bone spicules outside the macular area; Figure 1), fluorescein angiography (FA), full-field electroretinography (ERG), and electro-oculography (EOG) from the medical records. Full-field ERG and EOG were performed according to the International Society for Clinical Electrophysiology of Vision (ISCEV) standards, except in eight patients who underwent ERG examination before the ISCEV regulations, according to a similar protocol [15]. When no ERG or EOG testing was performed in our hospital, electrophysiologic findings were collected from referring institutions (10 patients). The ERGs were classified on the basis of the amplitudes of the scotopic rod B wave, photopic B wave, and 30-Hz flicker. Amplitudes of these different ERGs were considered to be abnormal when their values were less than the mean 5% of our local standard values. The resulting ERG recordings were classified in four groups (normal ERG, reduced cone response, reduced rod response, and reduced responses of both photoreceptor populations).

Patients who were suspected of STGD1 before the microarray screening were further subdivided into two groups, the typical STGD1 group or the atypical STGD1 group. We considered four necessary clinical features of typical STGD1 to be the following: 1) onset of symptoms before the age of 25 years; 2) a fundus appearance of type I or type II [14]; 3) a dark choroid on FA [16]; and 4) a normal scotopic ERG. An age at onset before the age of 25 years was chosen on the basis of a study that found a mean age at onset of 15.8 years with a standard deviation of 9.6 years in 278 STGD1 families [17]. Darkness of choroid in FA was assigned by an experienced retinal specialist (C.B.H.) without knowledge of the other characteristics of the patient’s phenotype. Patients without sufficient information on these criteria for a particular analysis were excluded for that analysis.

## Results

Sixty-five patients were included in this study, 31 males (48%) and 34 females (52%). The mean age was 34.6 years (standard deviation 15.7) at the time of the microarray screening, with a range from 4 to 69 years. In 44 patients (67.7%) STGD1 was considered the most likely clinical diagnosis before microarray screening. Of the remaining patients, 18 patients (27.7%) were suspected to have arCRD, and in three patients (4.6%) arRP was the most likely diagnosis. Of the 44 patients with suspected STGD1, mutation screening with the *ABCA4* microarray allowed the identification of 52 (59%) of a total of 88 expected pathogenic alleles. DGGE led to the finding of 12 more mutations, resulting in an overall detection rate of 73%.

The group of suspected STGD1 patients consisted of 12 patients (27%) in whom one mutation was found, 23 patients (52%) in whom two mutations were found, and two patients (5%) in whom three mutations were identified. In the group of patients carrying two or more mutations, sufficient information was available for segregation analysis in three cases. In these cases, different mutations matched different alleles. On retrospective analysis, two suspected STGD1 patients in whom mutations were identified could be classified as arCRD. The diagnosis was not altered on the basis of the genotype data but it was based on the clinical picture of these patients. Of the remaining seven patients (16%) in whom no mutations were found, the diagnosis of STGD1 was retained in two patients after retrospective analysis of the clinical picture.

In 18 patients (28%), arCRD was considered the most likely clinical diagnosis before microarray screening. In these patients, the microarray detected eight (22%) of 36 possible pathogenic alleles. Four patients (22%) showed a heterozygous sequence variant, and two patients (11%) showed a compound heterozygous sequence variant. DGGE analysis identified one additional homozygous mutation, resulting in a detection rate of 28%. Retrospective analysis of the clinical picture in patients without *ABCA4* mutations did not alter the diagnosis of arCRD. Finally, no mutations were found in the three patients who had arRP as the most likely diagnosis before microarray screening.

In 26 suspected STGD1 patients (59%), sufficient data were available for a classification in either the typical or the atypical STGD1 group. Six patients (23%) could be classified into the typical and 20 patients (77%) into the atypical STGD1 group. The occurrence of the four defining features of typical STGD1 is also separately presented in Table 1. All 74 mutations that were identified are listed in Table 2. Thirty-three mutations (44.5%) were missense mutations. The group of truncating mutations consisted of five nonsense mutations, 14 splice-site mutations, and five frameshift mutations, corresponding with 6.75%, 19%, and 6.75% of the total, respectively. The 2588G>C mutation was found in 11% and may result in a deletion or an amino acid substitution [13]. Finally, the 5461–10T>C mutation was found in 12%. The precise mechanism of the pathogenicity of this mutation has not been elucidated [18]. Webster and coworkers found that this mutation was significantly enriched in an STGD1 cohort when it was compared with a non-STGD1 cohort [9]. Some authors proposed the likelihood of a linkage disequilibrium of 5461–10T>C with an unidentified pathologic *ABCA4* mutation [19].

**Table 1:** Occurrence of different typical clinical features of Stargardt disease in patients stratified by the number of discovered *ABCA4* mutations.

**Clinical features**	**0 mutations**	**1 mutation**	**2–3 mutations**	**Total of patients^1^**
Number of patients	7	12	25	44
Age at onset <25 years	2/7 (29%)	8/12 (67%)	20/25 (80%)	44
Fundus appearance I or II^2^	5/7 (71%)	9/12 (75%)	20/25 (80%)	44
Dark choroid at FA	3/7 (43%)	4/10 (40%)	17/21 (81%)	38
Normal scotopic ERG	5/6 (83%)	7/7 (100%)	14/16 (88%)	29
Typical Stargardt^3^	1/6 (17%)	1/7 (14%)	4/13 (31%)	26

This table shows the proportion of patients suspected of Stargardt disease with typical clinical features of this disease. Results are separately shown for the patients with no, one, and more than one discovered *ABCA4* mutations. Abbreviations in the table are: *ABCA4*, ATP-binding cassette transporter gene *ABCA4*; FA, fluorescein angiography; ERG, full-field electroretinography. Superscript one indicates that patients from whom sufficient information was available were included in the analysis; superscript two indicates according to Fishman’s three-class system [14]; superscript three indicates typical Stargardt disease according to the occurrence of the four above mentioned clinical features.

**Table 2:** Discovered mutations in the *ABCA4* gene in the patients included in this study

**Nucleotide change**	**Effect**	**Alleles**	**References**
**Mutations already included in the *ABCA4* microarray**			
c.286A>G	p.Asn96Asp	2	[25]
c.656G>C	p.Arg219Thr	1	[10]
c.740A>T	p.Asn247Ile	1	This study*
c.768G>T	splice site	7	[13]
c.899C>A	p.Thr300Asn	1	[14]
c.1805G>A	p.Arg602Gln	1	[9]
c.1822T>A	p.Phe608Ile	2	[13]
c.1853G>A	p.Gly618Glu	1	[19]
c.1938–1G>A	splice site	1	[26]
c.2588G>C	p.DelGly863/Gly863Ala	8	[13]
c.2919del exons20–22	deletion/frameshift	2	[13]
c.3335C>A	p.Thr1112Asn	1	[13]
c.3874C>T	p.Gln1292X	1	This study*
c.3899G>A	p.Arg1300Gln	1	[27]
c.4297G>A	p.Val1433Ile	1	[17]
c.4462T>C	p.Cys1488Arg	1	[17]
c.4506C>A	p.Cys1502X	1	This study*
c.4539+1G>T	splice site	1	[28]
c.4774+1G>A	splice site	1	[1]
c.5161–5162delAC	p.Thr1721fs	1	[27]
c.5337C>A	p.Tyr1779X	1	This study*
c.5461–10T>C	unknown	9	[9]
c.5537T>C	p.Ile1846Thr	1	[13]
c.5693G>A	p.Arg1898His	1	[1]
c.5715+5G>A	splice site	2	[28]
c.5882G>A	p.Gly1961Glu	10	[1]
c.6088C>T	p.Arg2030X	1	[14]
c.6089G>A	p.Arg2030Gln	1	[9]
c.6238–6239delTC	p.Ser2080fs	1	[29]
c.6529G>A	p.Asp2177Asn	1	[1]
**New mutations found with DGGE analysis**			
c.303+4A>C	splice site	1	
c.872C>T	p.Pro291Leu	1	
c.2906A>G	p.Lys969Arg	1	
c.2947A>G	p.Thr983Ala	1	
c.3233G>A	p.Gly1078Glu	1	
c.3305A>T	p.Asp1102Val	1	
c.4353+1G>A	splice site	1	
c.5113C>T	p.Arg1705Trp	1	
c.5762_5763dup	p.Ala1922fs	1	
c.6411T>A	p.Cys2137X	1	
Total		74	

Mutations are designated by their nucleotide change, followed by their effect on the protein and the number of alleles that were found with the mutation. References are shown for mutations that have been earlier reported. Abbreviations in the table are: *ABCA4*, ATP-binding cassette transporter gene *ABCA4*; DGGE, denaturing gradient gel electrophoresis. The asterisks indicate the mutations that were included in the microarray, but to our knowledge, have not been reported before.

Fourteen different mutations were found that, to the best of our knowledge, have not been reported before (Table 2). Ten of them are mutations which were not included in the microarray at the time of analysis. They were found with DGGE analysis and were judged to be pathogenic according to our research protocol. First, the 6411T>A mutation, is predicted to result in a premature stop codon at amino acid number 2137. Second, there are two new mutations that affect the splicing of the RNA. Both 303+4A>C and 4352+1G>A are located in the consensus sequence of the intronic splice donor site. Therefore, these mutations are likely to be pathogenic. This is also the case with the 5762_5763 dup mutation as it results in a frameshift mutation and therefore disturbs the reading frame in the mRNA. Finally, DGGE found six new missense mutations. These mutations give rise to a new amino acid with different polarity or charge, except for the 2906A>G mutation, which results in a substitution of a lysine to an arginine. These amino acids have a net positive charge located at the amino group. An explanation for the pathogenicity of this mutation may be that this amino acid is located in a highly conserved region of the ABCR protein.

In two cases, DGGE analysis in our laboratory revealed a mutation that was also included in the microarray, whereas microarray results were normal for screening of this mutation.

## Discussion

For an evaluation of the *ABCA4* microarray in daily clinical practice, it is useful to look at the consequences of the screening results. The classification of patients suspected of STGD1 into a typical and an atypical STGD1 group (according to our definition) revealed that only 31% of patients with two or three discovered *ABCA4* mutations met all typical criteria of Stargardt disease (Table 1). On the other hand, in four out of six patients in the typical STGD1 group (67%), two or more *ABCA4* mutations were found. A limitation of this observation is that only 26 patients (59%) of all patients suspected of STGD1 underwent all examinations that were required to classify patients in the typical or atypical STGD1 group. When looking separately at the different defining features of typical STGD1 in Table 1, we conclude that only an age at onset before 25 years and a dark choroid on FA are more prevalent in patients with more discovered mutations than in patients with less or no discovered mutations in the *ABCA4* gene. The most striking is the difference in age at onset, which was younger than 25 years in 80% of patients with two or three discovered *ABCA4* mutations, in 67% of patients with one discovered mutation, and in only 29% of patients where no mutations were found. Furthermore, a dark choroid on FA was present in 81% of patients with two or three discovered *ABCA4* mutations, in 40% of patients with one discovered mutation, and in 43% of patients where no mutations were found. The latter rates differ from an earlier study where a dark choroid was found in only two of 12 patients (17%) with two discovered mutations and five of 17 patients (29%) with one discovered mutation [14]. This may be due to the lower number of patients in that study as well as a difference in clinical judgment of the FA.

Two suspected STGD1 patients in whom mutations were identified could retrospectively be classified as arCRD patients. These patients received additional examinations (e.g., full-field ERG) and routine follow-up as standard medical care. As a result, the diagnosis could change to arCRD, for instance as the disease progressed to a more panretinal dysfunction [20]. No mutations were found in seven of the 44 patients with suspected STGD1. The diagnosis was changed on retrospective analysis of the clinical characteristics in two of these cases. In one case, the diagnosis had changed to multifocal pattern dystrophy simulating STGD1/fundus flavimaculatus, after a mutation was detected in the *peripherin/RDS gene*. This patient was also described as C-III:2 in a study by Boon and colleagues [21]. In the other case, the absence of *ABCA4* mutations led to the retrospective diagnosis of central areolar choroidal dystrophy. The diagnosis of three other patients was still uncertain after the microarray screening because the clinical picture together with the negative results of the screening were too atypical for a certain diagnosis. In the remaining two patients in whom the diagnosis STGD1 was retained, one had a different clinical picture. Typical macular lesions were found accidentally, and the patient did not experience visual loss at the age of 42. However, she had a dark choroid and a normal ERG. This may be a form of asymptomatic late-onset STGD1 [20].

In our routine clinical DNA diagnostics setting, we obtained an *ABCA4* mutation detection rate of 59% with the microarray only and 73% with additional DGGE analysis in patients who were suspected of STGD1. These detection rates correspond with the detection rates found by other studies [10,22]. In the study of Maia-Lopez and coworkers, a detection rate of 55% that was obtained with the microarray alone was elevated to 67% with additional denaturing high-performance liquid chromatography (dHPLC) [22]. In arCRD, we have found a detection rate of 22% with the microarray alone; the detection rate was 28% with additional DGGE. In RP patients, we found no mutations. Compared with an earlier study, which found a detection rate of 33% in arCRD and 5.6% in arRP with the microarray only, our detection rates are low [19]. However, in another study at least one mutation was found using dHPLC in 23.6% of the patients with arCRD or sporadic CRD. No significant difference was found between the arCRD cases and the sporadic CRD cases. When the detection rate is calculated in the same way as in our study, a mutation was discovered in 19 of the 110 alleles (17%) [23]. Overall, we can not draw strong conclusions from our data regarding arCRD and arRP because mutations were screened in only 18 arCRD patients and three arRP patients.

In our study, microarray screening missed two mutations that had already been tested by the microarray, leading to 3% false-negative results. A possible explanation may be that the signals of the microarray for these mutations were too weak. False-positive results were not found in this study by direct sequencing. In a recent study by Aguirre-Lamban and coworkers, STGD1, arCRD, and arRP patients in whom the *ABCA4* microarray identified none or only one allele, were further evaluated by dHPLC and multiplex ligation-dependent probe amplification (MLPA). Both false-negative and false-positive rates for the microarray were 1.6%. A mutation detection rate of 43.5% for the microarray was increased to 64.5% by dHPLC alone [24]. We no longer use DGGE to search for unrevealed mutations. Instead, we use direct sequencing, which is now the gold standard.

The total carrier frequency of mild, moderate, or severe *ABCA4* mutations has been estimated to be one in 17 [13]. Therefore, we cannot exclude that patients in whom we have found only one mutation are coincident carriers of an *ABCA4* mutation (i.e., that this mutation is not involved in the disease of a patient). However, the mutations in the patients in this study may be assumed to be involved in the disease based on the clinical pictures of these patients. In addition, we have also determined the pathogenicity of all discovered variants according to the criteria of Cotton and coworkers [12].

In the last decade, it has become feasible for the general ophthalmologist to obtain molecular genetic support for a clinical diagnosis, for instance of STGD1. In daily clinical practice, in addition to confirmation of the diagnosis in typical STGD1, *ABCA4* microarray screening is usually requested to strengthen the diagnosis when the disease is atypical. Most STGD1 patients in our study, even those in whom two *ABCA4* mutations were found, did not meet all typical disease criteria. In addition, our study supports the view that the efficiency and accuracy of the *ABCA4* microarray are directly dependent upon the clinical features of the patients who are screened. DGGE analysis can raise the detection rate from 59% to 73% in patients who are suspected of STGD1 disease and may find mutations that are undetected by the microarray. When no mutations are found, this does not entirely exclude the diagnosis of STGD1 but may lead to a change in diagnosis on later follow-up.
